# The Pathological Mechanism Between the Intestine and Brain in the Early Stage of Parkinson's Disease

**DOI:** 10.3389/fnagi.2022.861035

**Published:** 2022-06-24

**Authors:** Runing Yang, Ge Gao, Hui Yang

**Affiliations:** Department of Neurobiology, School of Basic Medical Sciences, Capital Medical University, Beijing Key Laboratory of Neural Regeneration and Repair, Beijing Key Laboratory on Parkinson's Disease, Key Laboratory for Neurodegenerative Disease of the Ministry of Education, Beijing Institute of Brain Disorders, Collaborative Innovation Center for Brain Disorders, Beijing, China

**Keywords:** Parkinson's disease (PD), α-synuclein (α-syn), propagation, vagus nerve, immune inflammation, microbial-intestinal-brain axis

## Abstract

Parkinson's disease (PD) is the second most common chronic progressive neurodegenerative disease. The main pathological features are progressive degeneration of neurons and abnormal accumulation of α-synuclein. At present, the pathogenesis of PD is not completely clear, and many changes in the intestinal tract may be the early pathogenic factors of PD. These changes affect the central nervous system (CNS) through both nervous and humoral pathways. α-Synuclein deposited in the intestinal nerve migrates upward along the vagus nerve to the brain. Inflammation and immune regulation mediated by intestinal immune cells may be involved, affecting the CNS through local blood circulation. In addition, microorganisms and their metabolites may also affect the progression of PD. Therefore, paying attention to the multiple changes in the intestinal tract may provide new insight for the early diagnosis and treatment of PD.

## Introduction

Parkinson's disease (PD) is the second most common neurodegenerative disease (Grosso Jasutkar et al., 2022). PD is characterized mainly by motor disorders such as tremor, muscle stiffness, motor retardation and gait impairment (Nalls et al., 2014). These motor symptoms appear mainly in the middle and late stages of the disease (Machado et al., 2016; Wiratman et al., 2019). In addition, PD also shows non-motor symptoms (NMS) in the prodromal stage (Hussein et al., 2021), such as gastrointestinal motility disorder, decreased sense of smell, and rapid-eye-movement (REM) sleep behavior disorder. NMS are also a major cause of disability during the clinical stages of PD and progress all through PD (Poewe et al., 2017). Gastrointestinal dysfunction is the main non-motor symptom in patients with PD. The clinical manifestations are dysphagia, delayed gastric emptying and constipation, among which constipation is the most common symptom (Knudsen et al., 2017; Ahn et al., 2021). Clinical reports have shown that nearly 30% of patients with PD develop constipation 20 years before motor symptoms appear (Savica et al., 2009; Schapira et al., 2017; Manfredsson et al., 2018). About 70–80% of PD patients suffer from constipation, which is four times higher than normal people of the same age and sex (Hurt et al., 2019). When motor symptoms occur in PD patients, the degenerative changes are accompanied by a loss of 40–60% of the nigral dopamine neurons (D'Andrea et al., 2019), and an 80% reduction in striatal dopamine (Rabiei et al., 2019). At this time, PD is at an irreparable stage (Savica et al., 2018). Therefore, detecting the early NMS of the disease and providing intervention as early as possible, would be an effective means of treating or delaying the development of the disease.

Several lines of evidence suggest a close relationship between the intestinal nerve and the central nervous system (CNS) during the pathogenesis of PD. Braak and collaborators initially hypothesized that the gut-brain axis is involved in PD (Braak et al., 2006). Pathogens in the environment could pass through the intestinal epithelium and induce misfolding and accumulation of α-synuclein in specific neurons of the intestinal nerve, and this agent may mediate the propagation of pathology. But this connection still remains a hypothesis and it will be very difficult to prove in humans or will remain controversial. Recently, it was discovered that patients who had undergone a total vagotomy due to peptic ulcers had a lower incidence of PD than people who had not undergone this procedure or who had a selective partial vagotomy (Svensson et al., 2015). However, another study discussed clinical evidence that the case is not very clear (Tysnes et al., 2015). In addition, experimental evidence in rodents showed that the injection of the aggregated form of α-synuclein into the intestinal wall could promote the accumulation of endogenous α-synuclein (Kim et al., 2019; Van Den Berge et al., 2019). These evidence indicated that α-synuclein aggregates contributed to the accumulation of aggregated α-synuclein in various brain regions through vagus nerve transmission. This transmission was time-specific and region-dependent (Kim et al., 2019). The gastrointestinal tract is innervated by parasympathetic vagal and sympathetic non-vagal pathway. Structures along these pathways also show α-synuclein pathology (Braak et al., 2007). This means that the propagation of α-synuclein pathology *via* sympathetic nerves, mediated by the intermediolateral nucleus of the spinal cord, provides an additional potential route to the brain (Van Den Berge et al., 2021). These studies have confirmed that colonic lesions triggers the CNS lesions. However, a seminal work provided detailed studies in postmortem tissue that the “body-first” hypothesis is not the ultimate disease mechanism for PD (Beach et al., 2021), indicating that the relationship between the CNS and the enteric nervous system (ENS) might be bidirectional (Garrido-Gil et al., 2018). The nervous systems of the brain and the intestine are interlinked and this gut-brain axis can play a critical role pathogenesis and progression of PD.

The intestinal tract is the largest and longest immune organ in mammals (Liu et al., 2018). Therefore, the mucosal immune barrier and abundant immune cells in the intestinal tract may play an important regulatory role in gastrointestinal inflammation in PD. Studies have confirmed that intestinal nerve-derived IL-18 signals can control intestinal immunity and have a far-reaching impact on the mucosal barrier (Jarret et al., 2020). Gastrointestinal infections are associated with an increased risk of PD (Nerius et al., 2020). A recent study showed that α-synuclein is required for normal immune function, such as the development of a normal inflammatory response to bacterial peptidoglycan introduced into the peritoneal cavity as well as antigen-specific and T cell responses following intraperitoneal immunization (Alam et al., 2022). These indicate the presence of information exchange between the ENS and the immune system. Enteric glial cells (EGC) around intestinal neurons have closely interaction with intestinal neurons (Clairembault et al., 2015b). Intestinal neurons and glial cells may be targets for the treatment of intestinal inflammatory diseases, such as inflammatory bowel disease (IBD), *via* regulating the barrier function or immune response (Puzan et al., 2018; Li et al., 2020). Some studies have confirmed that the expression of pro-inflammatory cytokines and glial markers was increased in colon biopsies of patients with PD (Devos et al., 2013).

Intestinal innate immunity is also involved in the follow-up process of the T cell immune response. The antigen-presenting cells, such as dendritic cells (DCs), may take up previous inclusion bodies and then present the major histocompatibility complex (MHC) peptides derived from this process. The MHC peptides can be recognized by specific T-cell receptors (TCRs) on T cells. This triggers activation of the adaptive immune cells in the intestine, such as T cells, causing chronic inflammation (Campos-Acuña et al., 2019). At the same time, regulatory T (Treg) cells or other immune cells can also be activated to play an anti-inflammatory role through the dopamine pathway (Levite, 2016; Xue et al., 2018; Campos-Acuña et al., 2019) and short-chain fatty acid pathway (Zeng and Chi, 2015; Yuan et al., 2021). A variety of immune cells acquire immunophenotype in the intestinal tract and are transported to the CNS through abundant local blood flow, which affects the central immune response (Korn and Kallies, 2017). T cells are a “double-edged sword” and these cells undoubtedly add a new possibility to the pathogenic and therapeutic mechanisms of PD.

The intestinal tract has close contact with the external environment. Changes in the microflora in the intestinal cavity may be closely related to immune inflammation and the aggregation of pathological α-synuclein (Sampson et al., 2016). Changes in the intestinal flora can also affect the integrity of the blood-brain barrier (Rutsch et al., 2020). Short-chain fatty acids (SCFAs), the metabolites of intestinal flora, have multiple protective effects. Changes in the intestinal flora and its metabolites are indispensable factors affecting the occurrence and development of PD (Cholan et al., 2020).

While there is also the hypothesis of “brain-first” for PD and the evidence for that is similar and based on neuropathology in humans, this review will focus on the “body-first” hypothesis with an emphasis on the role of the microbiome and immune pathways.

## Pathophysiological Mechanism of Constipation in PD

There are various gastrointestinal symptoms (GIS) in patients with PD, including dry mouth, drooling, dysphagia, constipation, and defecation dysfunction (Travagli et al., 2020). Clinical studies have confirmed that the symptoms of gastroparesis in patients with PD precede motor symptoms, although the prevalence rate between PD and the control group was not significantly different (Edwards et al., 1991; Cersosimo et al., 2013). Constipation and defecation dysfunction are the main pre-motor GIS of PD.

According to different diagnostic criteria of chronic constipation, the prevalence rate of constipation ranges from 24.6 to 63% (Stocchi and Torti, 2017). Constipation is one of the main and crippling NMS of PD. In this review, we will first discuss the pathophysiological mechanism of PD-related constipation.

### α-Synuclein Is Involved in the Pathophysiology of Intestinal Function

α-Synuclein is the major component of the intraneuronal Lewy bodies (LBs) (Grochowska et al., 2021) and Lewy neurites (LNs) (Ehgoetz Martens and Lewis, 2017), the pathological hallmarks of PD (Koprich et al., 2010). Pathological aggregation of α-synuclein in gastric and colonic neurons has been detected in autopsies from patients with advanced PD (Wang et al., 2012). These neuroanatomical changes observed in patients with PD suggest that there is an abnormal accumulation of α-synuclein in the gastrointestinal system, which may play a role in the development of the gastrointestinal pathology in PD.

α-Synuclein is widely expressed in the brain and is thought to have a variety of functions, including regulating the release of neurotransmitters and vesicular circulation of central synapses (Burré et al., 2018). However, little is known about the physiological and pathological functions of α-synuclein in the peripheral nervous system. Studies on the gut of humans and guinea pigs have found that α-synuclein is expressed in the cell bodies of some intestinal neurons, especially in the varicosities and terminals of cholinergic neurons, and has an immune response to vesicular acetylcholine transporter (Vacht) (Sharrad et al., 2013). Some studies have confirmed that α-synuclein can regulate the development of cholinergic neurons (Swaminathan et al., 2019). It is not clear how α-synuclein affects and regulates intestinal function.

Studies on α-synuclein pathology have shown that α-synuclein pathology, induced in the α-synuclein virus overexpression model and prefabricated fibril (PFF) model, leads to abnormal gastrointestinal motility in the ENS of rats and non-human primates (Manfredsson et al., 2018). Human A53T α-synuclein transgenic mice have gastrointestinal disorders in the early stage (<6 months), insufficient intestinal peristalsis and decreased motor response of the longitudinal and circular muscle layer of the colon (Rota et al., 2019). Verification on a variety of animal models showed a correlation between intestinal α-synuclein pathology, reduced cholinergic function and prolonged gastric transit time in PD (Van Den Berge et al., 2021; Van Den Berge and Ulusoy, 2022). While it has been inferred that the possible mechanism of constipation might be the weakening of cholinergic transport in the ENS by α-synuclein pathology, this remains to be further studied.

### Loss of Intestinal Neurons Leads to a Defecation Disorder

In PD, constipation is due to slower colonic transit or outlet dysfunction, or both (Stocchi and Torti, 2017). In either case, it may be related to an imbalance in the control of defecation by the intestinal nerve. The early involvement of intestinal neurons may explain the occurrence of constipation in early PD.

Previous studies have confirmed the loss of vasoactive intestinal peptide (VIP) neurons in the colon of patients with PD (Wakabayashi et al., 1993) and the loss of excitatory dopaminergic neurons in the colon of the 1-methyl-4-phenyl-1,2,3,6-tetrahydropyridine (MPTP) model (Anderson et al., 2007). Both showed a deficiency in the relaxation function of the colonic smooth muscle, which may be closely related to the occurrence of constipation. Some studies have also confirmed a large loss in the number of dopamine neurons in the colonic myenteric plexus in patients with PD (Singaram et al., 1995). In contrast, recent studies have detected Lewy pathology in the colonic submucosal biopsies from PD patients (Shannon et al., 2012). However, no significant difference was found in the intermuscular neuron density between PD patients and the control group (Annerino et al., 2012). This may mean that the decrease in the intermuscular neurons may be the result of the gastrointestinal symptoms rather than the cause. This study also suggested that the neuropathology of the dorsal motor nucleus of the vagus nerve (DMV) and/or submucosal plexus is more likely than myenteric plexus injury to be the cause of the gastrointestinal motility disturbance associated with PD.

### Constipation and the Presence of Lewy Pathology in the ENS

Some studies have found that Lewy body dementia (DLB), another disease with the same Lewy body pathology as PD, also produces non-motor disorders (Fereshtehnejad et al., 2019). Literature statistics have shown that in DLB, α-synuclein aggregation appears earlier in the peripheral nerves than in the brain, such as the vagus nerve (86.7%), myenteric nerve plexus (86.7%), and cardiac sympathetic nerve (100%) (Gelpi et al., 2014). Constipation associated with Lewy bodies is called Lewy body constipation. Constipation in DLB may be more common than in PD (Sakakibara et al., 2019). Therefore, the current research on non-motor disorders in patients with PD should not be limited to PD but should include Lewy body disease and all neurodegenerative diseases.

## PD Pathology Spreads Along the Brain-Gut Axis Through the Vagus Nerve

**Figure 1:**
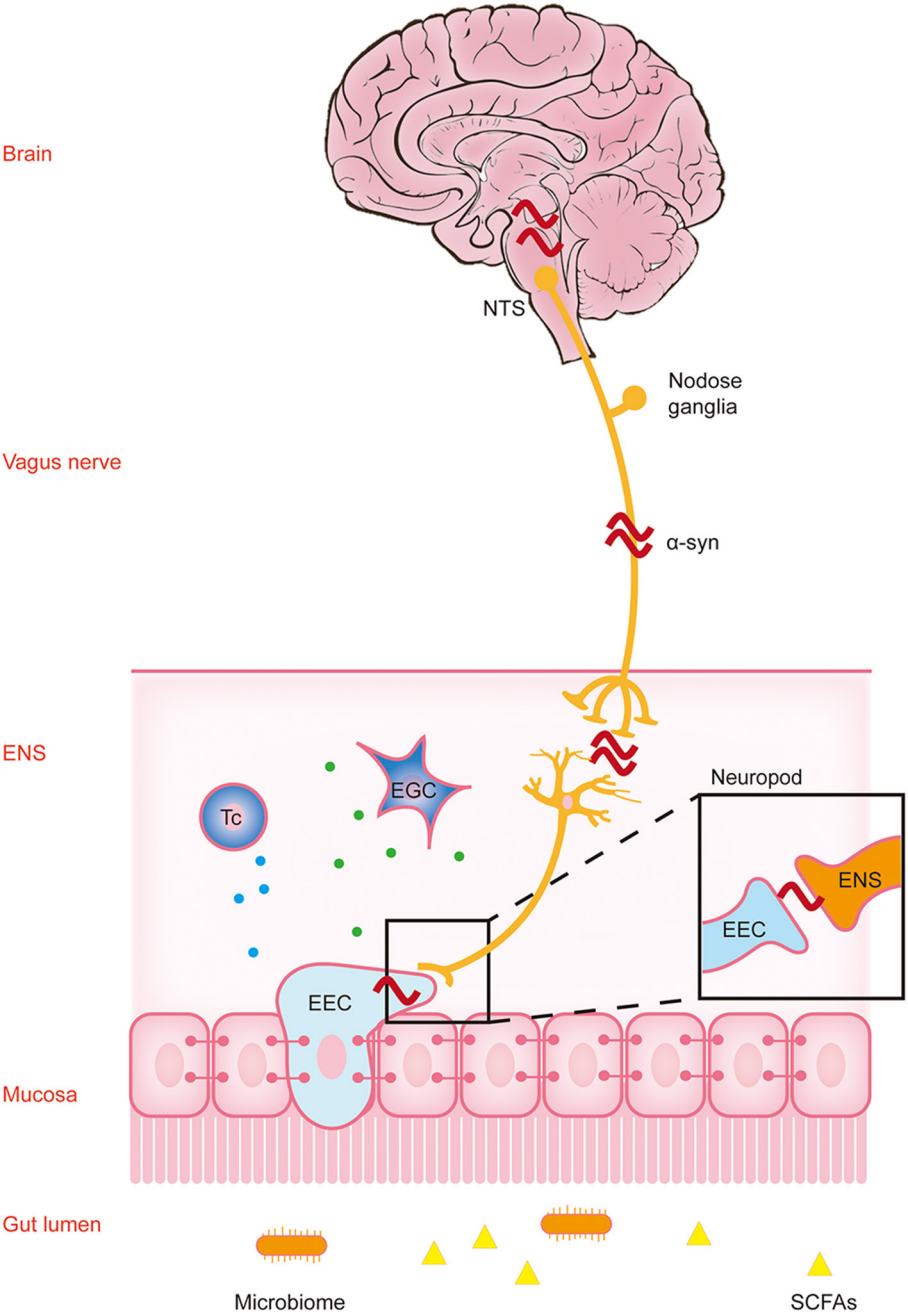
Parkinson's disease pathology spreads along the brain-gut axis through the vagus nerve. This figure reflects the “gut-first” hypothesis as well as the “brain-first” hypothesis was omitted. The environmental changes in the intestinal lumen may pass through the gastrointestinal wall and enter the myenteric neurons, triggering the formation of α-synuclein inclusions in the enteric nervous system (ENS). Parkinson's disease (PD) pathology was first found in the ENS, and this process may cause gastrointestinal dysfunction in the early stage of PD. The change in the lumen environment may lead to pathological changes in α-synuclein in enteric endocrine cells (EEC). Through the structure named “neuropods,” this signal is transmitted to the ENS, and induces the pathological changes in α-synuclein in the ENS. A variety of immune cells in the lamina propria may participate in this process through a variety of immune responses. Subsequently, α-synuclein ascends retrograde through the vagus nerve to the neurons in the dorsal motor nucleus of the vagus (DMV) in the brainstem, and finally reaches the substantia nigra pars compacta (SNpc), causing dopaminergic neuronal degeneration. At this time patients show typical motor symptoms of PD. NTS, nucleus tractus solitarius; α-syn, α-synuclein; Tc, T cells; EGC, enteric glial cells.

What we know so far is that constipation occurs in the early stage of PD and there is a variety of pathological changes associated with it. So, is there a relationship between early intestinal changes and the occurrence and development of CNS lesions in the later stage? Furthermore, does PD originate from the intestinal tract and spread to the CNS through a particular mode of transmission to cause pathological changes? Next, we will discuss this part of the content (Figure 1).

### Intestinal Pathology Occurs Earlier Than CNS Pathology in PD

Neurodegenerative diseases have always been regarded as central system diseases. Therefore, most studies have focused on the CNS. However, peripheral pathology is also closely related to the occurrence and development of CNS diseases. Braak et al. demonstrated that LBs are present in the neurons of the Meissner plexus of the stomach of patients with early stage PD, further supporting the hypothesis that the periphery, especially the ENS, is the origin of PD. The pathology of PD may start in the gastrointestinal tract and then spread to the brain through the vagus nerve. The findings by Braak and colleagues suggest that in the pathogenesis of PD, pathological alterations appear in the DMV and the ENS before they develop in the substantia nigra (Braak et al., 2006).

At present, this view is supported by pathophysiological evidence. It has been reported that α-synuclein inclusions appear in the ENS, glossopharyngeal nerve, and vagus nerve in the early stage of PD (Burré et al., 2018). Immunohistochemical detection in PD transgenic mice showed that a few months before the loss of striatal dopaminergic neurons, age-dependent α-synuclein-GFP was progressively expressed and accumulated in the Meissner and Auerbach plexus of the colon (Chen et al., 2018a). Some studies have shown that different forms of α-synuclein can be transmitted to the brain through the vagus nerve, which may be the mechanism of prion-like transmission of α-synuclein in PD and related diseases (Zhong et al., 2017; Kim et al., 2019; Liu et al., 2021a). This evidence all shows that the abnormal intestinal α-synuclein deposition is earlier than the occurrence of degenerative diseases of the CNS (Hilton et al., 2014).

### The Theory of Transmission of PD Pathology *via* the Vagus Nerve

The view that α-synuclein deposition in the intestine is earlier than in the CNS, and that Lewy pathology begins in the DMV has been supported. Moreover, the gastrointestinal system and the brain are anatomically connected through the vagus nerve. Although supported by the above theories, no studies have fully confirmed this process. The theory that PD begins in the intestinal tract and spreads by the vagus nerve is still widely debated (Gershanik, 2018).

The ENS is one of the earliest structures showing PD pathology, but the experimental results have not been confirmed in a large autopsy cohort study. It has also been shown that some patients show early pathology in the intermediolateral nucleus of the spinal cord (IML) and autonomic ganglia (data from large patient cohorts), also indicating a peripheral start of disease (Borghammer et al., 2021). α-Synuclein injected into the stomach of rats was retrogradely transported from the intestine to the brain through the vagus nerve (Kurnik et al., 2015). Another recent study confirmed that exogenous venereal α-synuclein injection could induce the accumulation of endogenous α-synuclein in the gastrointestinal tract and transmit it to the brain along the vagus nerve, causing corresponding pathological changes in various brain regions that finally lead to motor and cognitive impairment in the mice (Kim et al., 2019). A recent study indicated that propagation of α-synuclein pathology from the gut to the brain is more efficient in old vs. young wild-type rats, upon gastrointestinal injection of aggregated α-synuclein (Van Den Berge et al., 2021). However, overexpression of α-synuclein has also been proven to be transmitted from the brain to the ENS as a bidirectional pathway (Santos et al., 2019), which can not mean that the pathological changes in the brain must come from the intestinal tract. A study from Borghammer and Van Den Berge formulated two hypothesized PD-subtypes, a “body-first” subtype where pathogenic α-synuclein arises in the body and spreads to the brain, and a “brain-first” subtype where pathogenic α-synuclein arises in the brain and spreads to the body. Recent studies report new evidence in support of this hypothesis from newly diagnosed PD patients (Horsager et al., 2020; Borghammer et al., 2021; Knudsen et al., 2021) and animal models (Van Den Berge and Ulusoy, 2022). Moreover, the toxic changes of endogenous α-synuclein in the intestinal tract have not been solved. Therefore, the scientific question of whether and why the pathology of PD begins in the intestinal tract remains to be explored.

### EECs Are Involved in the Nerve Transmission Pathway

Enteric endocrine cells (EECs) are cells that sense the content of substances in the lumen, produce and release hormone/signal molecules, regulate a variety of physiological functions in the intestine and maintain homeostasis (Liddle, 2018). In recent years, the role of EECs in gut-brain/brain-gut communication has sparked people's interest (Ye et al., 2021). It was found that the cytoplasmic processes of cholecystokinin (CCK) and peptide tyrosine-tyrosine (PYY) cells are similar to axons, and their synaptic ends have been named “neuropods.” They contain a large number of secretory vesicles, many of which are distributed at the tip, indicating that they may guide the process of hormone secretion (Bohórquez et al., 2015). “Neuropods” contain intermediate filaments, are surrounded by glial cells, and appear to connect directly to nerves (including sensory nerve endings) (Latorre et al., 2016).

Intestinal nerves are not exposed to the intestinal lumen, so they are not directly affected by substances in the lumen. However, EECs have many neuron-like characteristics, which provide a possible way for intestinal luminal substances and intestinal nerves to communicate with each other. Rabies virus and mad cow disease prion can infect EECs and transfer to the intestinal nerve (Liddle, 2018). It has been confirmed that EECs express α-synuclein (Chandra et al., 2017), and that misfolded α-synuclein has the ability to transfer from nerve to nerve in a prion-like manner (Angot et al., 2012). Therefore, EECs may be involved in the neural transmission pathway in PD. α-Synuclein can misfold in EECs due to change in the intestinal environment and other factors. Through the prion-like characteristics and neuron-like properties of EECs, the misfolded α-synuclein may spread to the adjacent ENS and eventually spread to the brain through the vagus nerve pathway.

## PD Pathology Transfers to the CNS Through the Humoral Pathway

**Figure 2:**
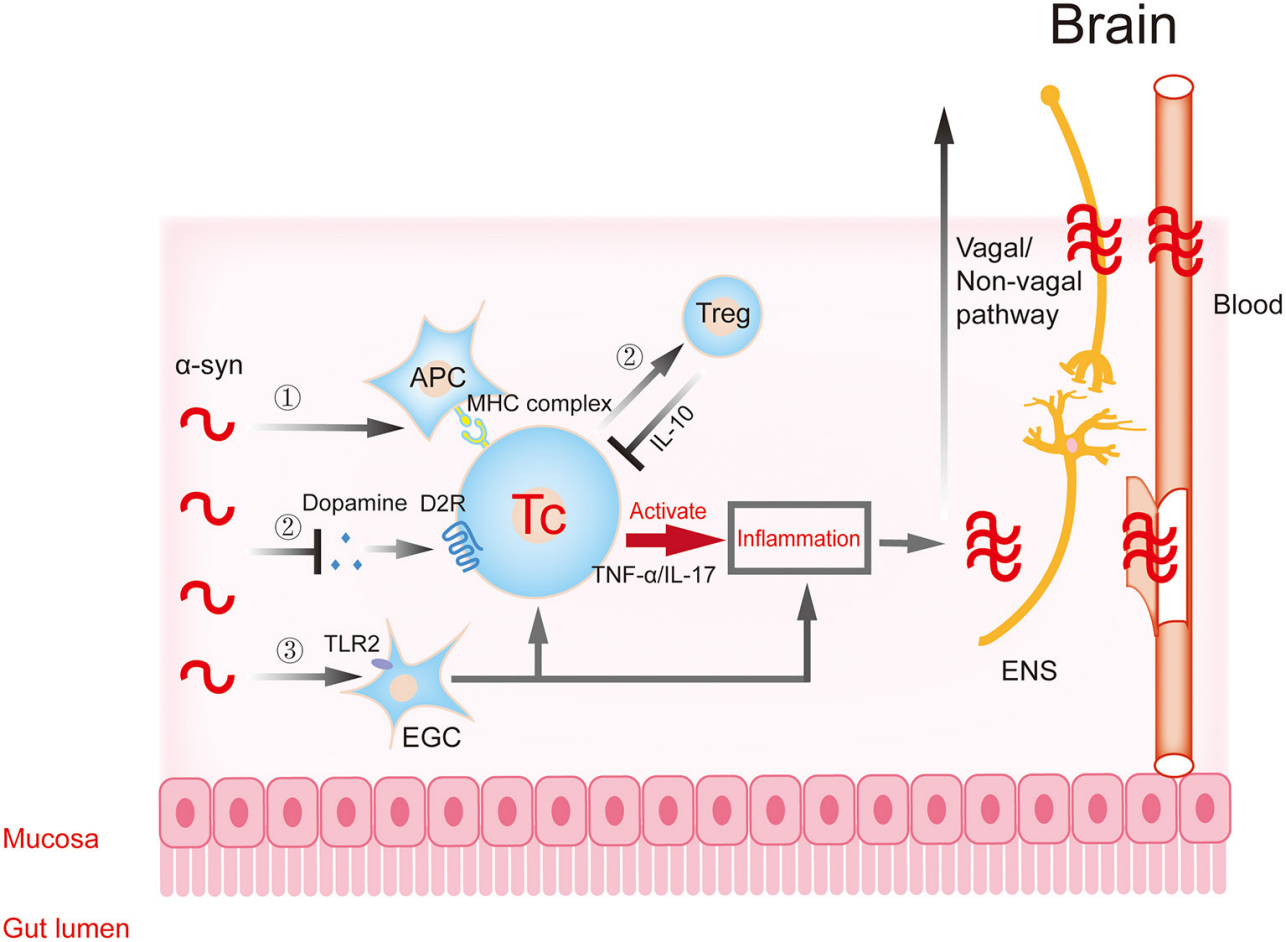
Gastrointestinal immune inflammatory response participates in the progression of α-synuclein. α-Synuclein may activate T cells to trigger downstream inflammatory responses through APCs (①) and EGCs (③), resulting in traumatic changes in intestinal structure and function. The inflammatory reaction further aggravates the aggregation and toxicity of α-synuclein. The excessive accumulation of α-synuclein in intestinal neurons can inhibit synaptic vesicle transport and reduce dopamine production, then inhibit the transformation of T cells to protective regulatory T (Treg) cells, and aggravate inflammation (②). Dopamine in this process participates in T cell protective regulation through the dopamine receptor. The pro-inflammatory or anti-inflammatory reaction caused by the above processes further aggravate the aggregation and toxicity of α-synuclein. Subsequently, these changes affect the central nervous system (CNS) through the dual pathways of nerve (vagal and non-vagal circuits) and blood circulation. α-syn, α-synuclein; APC, antigen presenting cell; EGC, enteric glial cells; ENS, enteric nervous system.

The transfer of gastrointestinal Lewy pathology to the CNS along the vagus nerve pathway in the initial stage of the disease leads to Lewy pathology and inflammation in the brain. However, the development of the disease may also be related to the peripheral intestinal tract. Because the intestine is the largest and longest immune organ of the body, and the intestinal cavity is in close contact with the external microenvironment, there are abundant immune cells and a variety of immune responses. Changes in intestinal permeability and continuous contact with a variety of antigens may trigger innate and acquired immune responses in the intestinal immune system, leading to gastrointestinal inflammation. Under normal circumstances, there is also an anti-inflammatory mechanism in the body. This immune balance is disrupted in the disease state. The activated immune cells are much more likely to affect the CNS through the humoral pathway and aggravate the level of pathology and inflammation in the brain. Therefore, the inflammatory changes caused by intestinal microecology and the regulation of immune cells involved in it provide new ideas for the potential risk factors and possible pathogenesis of PD (Figure 2, Table 1).

**Table 1:** Types of gastrointestinal immune cells associated with PD and their pro-/anti-inflammatory effects.

**Immune cell**	**Markers**	**Cytokines**	**Type of immune response**	**References**
Th1	CD3+CD4+IFN-γ+	IFN-γ, TNF-α, IL-17, IL-1, IL-2, IL-21	Pro- inflammation	Kaiko et al., 2008; Sulzer et al., 2017; Campos-Acuña et al., 2019
Th17	CD3+CD4+IL-17+			
Th2	CD3+CD4+IL-10+	IL-4, IL-5, IL-13	Anti-inflammation	González et al., 2015; Kustrimovic et al., 2018
Treg	CD4+CD25+Foxp3+	IL-10, TGF-β1	Anti-inflammation	Huang et al., 2017, 2020; Kustrimovic et al., 2018
DC	CD80+CD86+	IL-6, IL-12, IL-10	Present antigen	Pacheco et al., 2009
EGC (M1)	GFAP, SOX-10	TNF-α, IL-1β, IL-6	Pro-inflammation	Côté et al., 2011, 2015; González et al., 2015

*PD, Parkinson's disease*.

### Changes in Intestinal Wall Permeability

One of the important functions of the gastrointestinal tract is to act as a semi-permeable barrier, regulating the absorption of nutrients, ions, and water, and regulating host contact with a large number of dietary antigens and bacteria (Ménard et al., 2010). Intestinal permeability can be defined as a facility in which intestinal epithelial cells allow molecules to spread through passive diffusion, and it is an important index for evaluating the integrity of the mucosal barrier (Luissint et al., 2016). Several chronic autoimmune intestinal diseases, such as IBD, are associated with increased intestinal permeability (Chang et al., 2017). Case studies with patients with PD have confirmed that there is a significant increase in intestinal permeability, and increased exposure to intestinal bacteria and bacterial endotoxins in patients with early diagnosed PD (Karunaratne et al., 2020). The increased α-synuclein in intestinal biopsies was associated with high intestinal permeability (Oreja-Guevara et al., 2011; Schwiertz et al., 2018). Some literatures have detected changes in the distribution of the tight junction proteins ZO-1 and occludin in colonic mucosa samples of PD patients and decreased amounts of occludin (Clairembault et al., 2015a), which is related to changes in colonic permeability. The LPS mouse model has shown different intestinal permeability changes. That is, the increased intestinal permeability occurred mainly in the colon, while phosphorylated α-synuclein serine 129 was detected in the intermuscular neurons of the colon (Kelly et al., 2014).

Prolonged intestinal permeability dysfunction can lead to the translocation of bacteria (such as *Escherichia coli*) and bacterial products (such as lipopolysaccharide), which creates a pro-inflammatory environment and increases the burden of oxidative stress on the ENS. Various factors contribute to the appearance of pathological α-synuclein in the gastrointestinal tract and make the immune cells acquire an antigenic phenotype. Therefore, intestinal leakage in patients with a genetic susceptibility to PD may be a key early step in promoting the pro-inflammatory/oxidative environment. Combined with the transmission theory of PD, this change in the microenvironment would contribute to the initiation and/or progression of PD.

### Regulation of Gastrointestinal Inflammation by the Innate Immune Response

Crohn's disease (CD) and ulcerative colitis (UC) are two IBD. UC involves the colon and rectum, while CD involves the small intestine and colon (Chang, 2020). Coincidentally, IBD and PD have some common characteristics, including several common risk genes, such as LRRK2 (Hui et al., 2018). In recent years, several studies have reported the causal relationship between IBD and PD (Lin et al., 2016; Peter et al., 2018; Brudek, 2019; Kishimoto et al., 2019; Zhu et al., 2019; Rolli-Derkinderen et al., 2020; Wan et al., 2020; Lee et al., 2021). Therefore, the two diseases are often compared.

It has been confirmed that initially α-synuclein aggregation and subsequently Lewy body generation occur in neurons such as the olfactory bulb and gastrointestinal tract that are exposed to adverse environmental factors (Del Tredici and Braak, 2016). A study using endoscopic biopsy from children with intestinal inflammation found a significant correlation between the level of α-synuclein accumulation in the ENS and the degree of intestinal inflammation (Stolzenberg et al., 2017). Recent literature has determined the capacity of the appendix to modify PD risk and influence pathogenesis (Gray et al., 2014; Killinger et al., 2018; Gordevicius et al., 2021). Lewy bodies stimulated Toll-like receptor 2 (TLR2) and Toll-like receptor 4 (TLR4) on local glial cells to induce NF-κB activation through Toll-like receptor (TLR) signaling pathway, thus inducing initial inflammation in the microenvironment (González et al., 2015). The EGC population is species specific and as complex as CNS glia (Grundmann et al., 2019). Some literature has hypothesized that the inflammatory mediators produced by EGC may damage the surrounding tissue, and induce and aggravate the misfolding and accumulation of α-synuclein in the intestinal nerve (Fellner and Stefanova, 2013).

At present, there is sufficient evidence that innate immune cells are involved in PD-related gastrointestinal inflammation. Through the analysis of colon biopsies from patients with PD, it has been determined that pro-inflammatory cytokines (TNF-α, IF-γ, IL-6, and IL-1β) and glial cell markers GFAP and SOX-10 are significantly increased in patients with PD (Devos et al., 2013). Recent findings demonstrated that chronic colitis promotes parkinsonism in genetically susceptible mice, and TNF-α plays a detrimental role in the gut-brain axis of PD (Lin et al., 2021).

The interaction between intestinal inflammation and intestinal nerves has also been reported. It has been found that partial knockout of M1 monocytes has a neuroprotective effect on the myenteric plexus in the MPTP model, but has no protective effect on basal ganglia (Côté et al., 2015). Moreover, the ENS provides a key link in the innate immune response, which is not only important for coordinating mucosal barrier homeostasis, but also for combating invasive bacterial infections. Therefore, intestinal nerve damage caused by inflammation may further aggravate local inflammation (Jarret et al., 2020).

How local gastrointestinal inflammation in PD affects the follow-up progress of the disease in the CNS will be summarized in 4.4.

### Regulation of Gastrointestinal Inflammation by the Acquired Immune Response

Innate immunity may play an initiating role in gastrointestinal inflammation. In addition to innate immune inflammation, innate immunity can also participate in subsequently acquired immune inflammation. Acquired immunity plays the role of inflammatory cascade amplification and anti-inflammatory response, resulting in follow-up regulation of inflammation. Acquired immunity plays an important role in both neurodegenerative diseases and IBD (Table 2). Early studies showed that while B cells were not significantly increased, T cells were significantly increased in the postmortem brains of patients with PD and brains of the MPTP model. A decrease in dopaminergic cell death induced by MPTP was observed in CD4-deficient mice (Brochard et al., 2009). CD4^+^ T cells contribute to neurodegeneration in Lewy body dementia (Gate et al., 2021). This suggests that T cells, especially CD4^+^ T cells, may be involved in the pathogenesis of PD (Chen et al., 2019) or other neurodegenerative disease.

**Table 2:** Changes of immune cells in neurodegenerative diseases/IBD.

**Disease**	**CNS immunity**	**Peripheral immunity**	**Reference**
AD	Microglia lost phagocytic ability	Th1 cells↓, Treg cells↑	Baruch et al., 2015; Schwartz and Deczkowska, 2016
PD	Microglia (M1) activation, CD4^+^/CD8^+^↓	Th1 and Th17 cells↑, Treg cells↓	González et al., 2015; Moehle and West, 2015; Baird et al., 2019
MS	Proinflammatory, exacerbate disease	Th1 and Th17 cells↑, Treg cells↓	Yamasaki et al., 2014; Schwartz and Deczkowska, 2016; Quinn and Axtell, 2018
CD	Microglia activation	Th1/Th2↑, Th17 cells↑, Treg cells↑	Li et al., 2016; Kishimoto et al., 2019; Kredel et al., 2019
UC	Microglia activation	Th1/Th2↓, Th17 cells↑, Treg cells↑	Li et al., 2016; Kishimoto et al., 2019; Kredel et al., 2019

*IBD, inflammatory bowel disease; AD, Alzheimer's disease; PD, Parkinson's disease; MS, multiple sclerosis; CD, Crohn's disease; UC, ulcerative colitis; ↑, increase; ↓, decrease*.

α-Synuclein can be an antigenic substance. It has been reported that the peptides of its two regions (Tyr39 and phosphorylated Ser129 region) can be used as antigenic epitopes to stimulate infiltrating antigen-presenting cells (APCs) (that is, monocytes/macrophages and DC). These cells capture and receive stimulation through the TLR signaling pathway, then process Lewy bodies into suitable small peptides, before forming Lewy body-specific MHC-II antigens (Sulzer et al., 2017). Then, APCs present MHC-II antigen to naive CD4^+^ T cells (González et al., 2015; Sulzer et al., 2017). CD4^+^ T cells activate, proliferate, and differentiate into Th1 and Th17 cells, infiltrate into the lamina propria of the colon, and release IFN-γ, IL-17, and other inflammatory mediators that recruit and stimulate neutrophils and macrophages, thereby inducing chronic inflammation in the intestinal mucosa (Dardalhon et al., 2008). Reactive oxygen species (ROS) induced by Th1 and Th17 immunization in local phagocytes promotes the further accumulation of α-synuclein in ENS neurons (González et al., 2015). This mechanism creates a cycle of increased toxicity of α-synuclein. Moreover, the intestinal inflammation of IBD is driven mainly by Th1 and Th17 cells of the CD4^+^ T cell subset (Granlund et al., 2013). A study defined that a compromised immune system increases the accumulation of pathological α-synuclein in the brain (George et al., 2021). This suggests that the pro-inflammatory responses of CD4^+^ Th1 and Th17 play an important role in gastrointestinal inflammation in both IBD and PD.

Regulatory T (Treg) cells are an inhibitory subtype of lymphocytes and play an important role in maintaining intestinal homeostasis (Chen et al., 2021). Treg cells can inhibit the inflammation induced by effector T cells (Th1 and Th17) in chronic UC models induced by T cells transferred to lymphocyte knockout mice. One of the main inhibitory mechanisms depends on the secretion of IL-10 by Treg cells (Powrie and Mason, 1990). IL-22 produced by Treg cells induces the expression of tight junction proteins (claudin1 and ZO-1) in epithelial cells, thereby reducing intestinal infiltration, increasing the integrity of the intestinal mucosal barrier and protecting it from intestinal inflammation (Fang et al., 2018). In the presence of chronic neuroinflammation in the CNS, there is evidence that peripheral immune cells can infiltrate the CNS through the damaged blood-brain barrier. It has been confirmed that the adoptive transfer of CD4^+^CD25^+^ Tregs into the MPTP model could reduce neuroinflammation and protect dopaminergic neurons in the substantia nigra compacta of the model mice (Huang et al., 2020). Dopamine can enhance the protective effect of CD4^+^ T cells or reduce the inflammatory damage of CD4^+^ T cells so as to inhibit inflammation (Contreras et al., 2016; Ahlers-Dannen et al., 2020).

To sum up, Lewy body-derived antigens may be an important target for T cell-mediated immunity. Inflammation induced by CD4^+^ T cells promotes the further accumulation of α-synuclein. The pro-inflammatory response mediated by Th cells and the anti-inflammatory response driven by Treg cells show the subsequent regulation of acquired immunity on the inflammatory process and represent the central process of PD and IBD, and dopamine may regulate this process.

### Local Gastrointestinal Inflammation Affects the CNS Through the Humoral Pathway

It has been reported that a variety of neurodegenerative diseases (Sweeney et al., 2018), including PD, exist in the presence of blood-brain barrier damage (Varatharaj and Galea, 2017). Similarly, a variety of animal models of PD, such as the rotenone model (Ravenstijn et al., 2012), MPTP model (Liu et al., 2017), and LPS model (Sweeney et al., 2018), also show different degrees of damage to the blood-brain barrier. This damage to the blood-brain barrier may be attributed to the ascending transmission of Lewy bodies along the vagus pathway. Lewy bodies accumulate in the brain to produce pro-inflammatory mediators, such as IL-1β, which activate microglia and related inflammatory cytokines to cause damage to the blood-brain barrier (Varatharaj and Galea, 2017; Gordon et al., 2018). Peripheral inflammatory factors can also cause damage to the blood-brain barrier. There is abundant blood flow in the intestinal tract. Innate immune cells or antigen-activated T cells and corresponding inflammatory factors obtained in the gastrointestinal tract can enter the blood circulation, pass through the damaged blood-brain barrier, and migrate to the brain parenchyma, leading to neuroinflammation and neurodegeneration (Chen et al., 2018b). This mechanism represents a vicious cycle: Inflammation may continue to aggravate the production of Lewy bodies and damage the blood-brain barrier in the brain, which in turn leads to the continuous aggravation of inflammation and progression of disease.

## Potential Key Role of the Intestinal Flora in the Pathogenesis of PD

The gastrointestinal tract of healthy people is inhabited by a wide variety of microorganisms called intestinal flora. Bacteria in the human gastrointestinal tract can form a large and complex ecosystem. These microbes are involved in almost all intestinal functions, affecting host metabolism, and behavior, neural circuits, hormone secretion, and immune response (Cox and Weiner, 2018). In recent years, with the introduction of the concept of microorganism-intestine-brain axis, it was shown that there is a two-way interaction between intestinal flora and the brain (Cryan et al., 2019). This may play an important role in neurological diseases, including anxiety disorder, depression (Foster and McVey Neufeld, 2013), autism, multiple sclerosis, PD (Elfil et al., 2020; Liu et al., 2021b), and Alzheimer's disease (Quigley, 2017; Srivastav and Mohideen, 2021).

The gastrointestinal microbiome is altered in PD and likely plays a key role in its pathophysiology. A study showed that compared with healthy controls, the abundance of *Prevotella* in the feces of patients with PD is decreased (Unger et al., 2016). This results in decreased intestinal mucus secretion, increased intestinal permeability, and increased local and systemic susceptibility to bacterial antigens and endotoxins, as well as a large amount of α-synuclein expression and misfolding. However, changes in intestinal flora can also affect the occurrence of PD. Intestinal gram-negative bacterial infection in mice can induce a decrease in dopaminergic neurons in the substantia nigra of PINK1–/– mice and produce PD-like behavioral changes, such as dyskinesia (Matheoud et al., 2019). In the intestinal tissue of germ-free mice, the activation of microglia is decreased, the content of pathological α-synuclein is also significantly decreased (Sampson et al., 2016). There are obvious changes in the intestinal flora in patients with PD. Therefore, gut dysbiosis has a significant potential as a therapeutic target in PD.

### Changes in Intestinal Flora Affect the Blood-Brain Barrier

Some studies have found that the intestinal flora can change the permeability of the blood-brain barrier in germ-free mice, which indicates that changes in the intestinal flora will affect the defense function of the blood-brain barrier (Rutsch et al., 2020). The expression of different types of TLR in brain endothelial cells can respond to bacterial cell wall components such as lipopolysaccharide (LPS) of gram-negative bacteria and lipoteichoic acid (LTA) of gram-positive bacteria, and directly affect the function of the blood-brain barrier (Tang et al., 2017). LPS can also induce other cell types to produce and release pro-inflammatory mediators, and thus regulates the function of the blood-brain barrier (Nagyoszi et al., 2010).

### Protective Effect of SCFAs in Intestinal Flora

SCFAs, such as butyrate, acetate and propionate, are produced by the fermentation of dietary fiber by intestinal microflora. After reaching the CNS through the blood circulation, SCFAs enhance the blood-brain barrier function by up-regulating the expression of tight junction protein in the blood-brain barrier. SCFAs also have neurotrophic and anti-inflammatory effects (Al-Asmakh and Hedin, 2015; Cholan et al., 2020). Reductions in fecal SCFAs but increased plasma SCFAs were observed in PD patients (Aho et al., 2021; Chen et al., 2022). SCFAs can also improve the dysfunctional blood-brain barrier in germ-free mice (Braniste et al., 2014), and it is also beneficial to the intestinal mucosal barrier (Chen et al., 2017).

SCFAs can up-regulate brain-derived nerve growth factor and glial cell line-derived neurotrophic factor. They can also protect dopaminergic neurons by activating the expression of G protein coupled receptors and inhibiting histone deacetylase (Abdel-Haq et al., 2019). In animal experiments, microglia in the brain of germ-free mice have shown immaturity and had almost no response to inflammatory stimuli (Erny et al., 2015). After supplementing with SCFAs, immature microglia routinely matured and could be activated to respond to inflammation and stimulation, suggesting that SCFAs have a neuroprotective effect (Scott et al., 2017).

SCFAs can also regulate immunity through a variety of mechanisms (Yao et al., 2022). G protein-coupled receptor (GPR)-mediated SCFA signaling can stimulate the differentiation of Treg cells and inhibit intestinal inflammation, or regulate the differentiation of Treg cells through epigenetic modification, which is helpful for the dynamic balance of immunity in the colon. At the same time, SCFAs promote the production of IL-10 by microbiota antigen-specific Th1 cells to limit the induction of colitis (Sun et al., 2018).

## Discussion

PD is the second most common neurodegenerative disease. The main clinical manifestations are dyskinesia and non-motor symptoms. Gastrointestinal dysfunction is the main non-motor symptom in patients with PD, among which constipation is the most common. Constipation symptoms may appear 20 years earlier than exercise symptoms, suggesting that PD may originate from the intestinal tract.

The occurrence of constipation may be closely related to the loss of intestinal neurons and the pathology of α-synuclein. At present, although evidence supports the view that α-synuclein deposition occurs earlier in the intestine than in the CNS, whether PD pathology originates from the periphery and then affects the CNS, or whether PD begins in the intestinal tract, is still widely debated. In recent years, studies on the pathogenesis of the microorganism-gut-brain axis in PD suggest that early changes in the intestinal flora and α-synuclein expression in intestinal nerves cause toxic and aggregation-like changes under inflammation. These changes affect the CNS through the dual pathways of nerve (vagal and non-vagal circuits) and blood circulation. This causes CNS damage and promotes disease progression. In this process, the anti-inflammatory responses of Treg cells, the T cell anti-inflammatory pathway mediated by dopamine, and the multiple protective effects of SCFAs, produce certain anti-inflammatory effects. These results enrich the theory of the intestinal origin of PD and provide theoretical support for the discovery of new therapeutic targets for PD.

In the prodromal stage of PD, the changes in neural or immune molecules in the gastrointestinal tract and peripheral blood may become biomarkers of PD and provide the basis for early diagnosis. Based on the microorganism-gut-brain axis hypothesis in PD, each part of it may become a potential therapeutic target. Early application of drugs and antibodies against gastrointestinal α-synuclein or immune cells may reduce the transmission of α-synuclein. Dietary or pharmacological interventions aimed at modifying the gut microbiota composition and enhancing the intestinal epithelial barrier integrity in PD patients or subjects at higher risk for the disease may delay disease progression. Cellular therapies using Treg cells are currently undergoing clinical trials for the treatment of autoimmune diseases, transplant rejection, and Treg cells have also been shown to have neuroprotective effects in mouse models of Alzheimer's disease.

In this review, we first focused on the possible mechanism of constipation in PD. Then, by analyzing the effects of intestinal changes on the CNS, we analyzed the involvement of the vagus nerve in the transmission of α-synuclein to the brain, as well as the pro-inflammatory and anti-inflammatory responses of congenital and adaptive immune cells. We also considered the anti-inflammatory and protective functions of dopamine. Finally, we analyzed how the flora and its metabolites participate in this process. Combining these findings suggest that intestinal lesions may be the origin of PD. Intestinal pathological changes may spread to the CNS through the vagus nerve, causing pathological changes in the brain and inflammation. This leads to damage of the blood-brain barrier, which occurs at the initial stage of the disease. However, the immune response activated by the intestinal tract is key to the subsequent vicious cycle: Intestinal immune response aggravates intestinal α-synuclein deposition; activated immune cells pass through the damaged blood-brain barrier through blood circulation. Both may cause CNS inflammation and irreversible neurodegeneration.

## Author Contributions

RY performed the literature review, drafted and reviewed the manuscript, and designed the figures. GG reviewed the manuscript. HY made critical revisions to the manuscript and provided study supervision. All authors contributed to the article and approved the submitted version.

## Funding

This work was supported by grant of National Natural Science Foundation of China (81870994).

## Conflict of Interest

The authors declare that the research was conducted in the absence of any commercial or financial relationships that could be construed as a potential conflict of interest.

## Publisher's Note

All claims expressed in this article are solely those of the authors and do not necessarily represent those of their affiliated organizations, or those of the publisher, the editors and the reviewers. Any product that may be evaluated in this article, or claim that may be made by its manufacturer, is not guaranteed or endorsed by the publisher.
